# Effects of different levels of controlled hypotension on regional cerebral oxygen saturation and postoperative cognitive function in patients undergoing total knee arthroplasty

**DOI:** 10.3389/fmed.2022.989341

**Published:** 2022-09-14

**Authors:** Yajuan Zhao, Chuanbo Zang, Shengjie Ren, Jianbin Fu, Ning Liu, Ziyu Zhou, Bao Lang

**Affiliations:** ^1^Department of Anesthesiology, Weifang People’s Hospital, Weifang, China; ^2^School of Anesthesiology, Weifang Medical University, Weifang, China; ^3^The 80th Group Army Hospital of the Chinese People’s Liberation Army, Weifang, China

**Keywords:** controlled hypotension, cerebral oxygen saturation, postoperative cognitive function, aging people, TKA

## Abstract

**Background:**

Controlled hypotension technique was usually used to reduce intraoperative bleeding, and it could improve visualization of the surgical field during total knee arthroplasty (TKA). However, inappropriate controlled hypotension, through reducing cerebral blood flow or cerebral perfusion pressure, may cause postoperative cognitive dysfunction (POCD), so it is important to identify the appropriate level of controlled hypotension. Objective: To investigate the effects of different levels of controlled hypotension on regional cerebral oxygen saturation and postoperative cognitive function in patients undergoing TKA.

**Methods:**

Patients meeting inclusion criteria were enrolled through preoperative visits and basic information was obtained. The patients were randomly divided into three groups: Group A, MAP was maintained at 90–100% of the baseline; Group B, MAP was maintained at 80–90% of the baseline; Group C, MAP was maintained at 70–80% of the baseline. The MAP, HR, and rSO_2_ were observed and recorded during the operation. The C-reactive protein (CRP), hemoglobin (Hb) and MMSE score at 1, 3, and 7 days after operation were recorded. SPSS25.0 was used for data analysis.

**Result:**

When the MAP had a decrease among the three groups, rSO_2_ did not decrease significantly, and none of the patients experienced POCD which was measured by MMSE. And there was no correlation between the decline in rSO_2_ and that in MAP.

**Conclusion:**

No POCD was experienced in the three groups, and we recommend that the controlled hypotensive target indicated by MAP was maintained at 70–80% of the baseline which not only decreases intraoperative bleeding and improve the quality of the surgical field, but also is still within safe levels.

## Graphical Abstract

Study flowchat. 
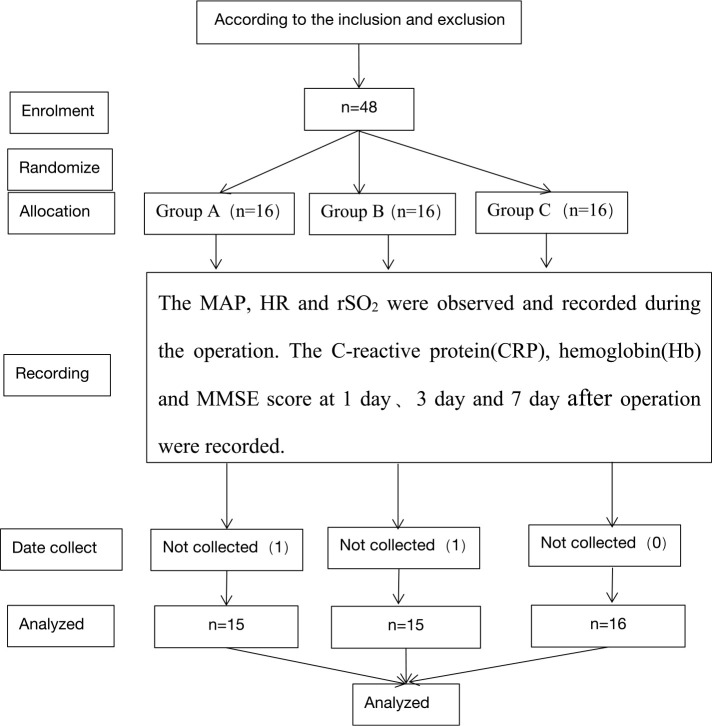

## Introduction

Total knee arthroplasty (TKA) is an effective method for the treatment of end-stage knee osteoarthritis. However, the large surgical wound and the amount of bleeding may seriously affect the prognosis. The traditional method is to use the tourniquet technique during surgery, and problems such as muscle and nerve injury caused by tourniquet are inevitable. Compared with tourniquet technique, controlled hypotension may have more advantages in TKA, which allows reduction of bleeding in the surgical field, minimization of blood loss, better visibility and therefore, it increases the surgeon’s comfort, reduces the surgery time and prevents complications.

Controlled hypotension is defined as the reduction of systolic blood pressure (BP) to 80–90 mmHg, and that of MAP to 50–65 mmHg or 30% lower than the baseline level (1) while still providing adequate oxygen delivery to vital organs. However, extreme hypotension may cause.

Organ insufficiency and subsequent ischemic injury of vital organs, especially the brain (2), which has potential risk to cognitive function. Therefore, we set the 30% decrease from the baseline MAP as the minimum levels to investigate the effects of different levels of controlled hypotension on regional cerebral oxygen saturation and postoperative cognitive function in patients undergoing TKA.

Postoperative cognitive dysfunction (POCD) is a common complication induced by anesthesia or surgery, which affects the concentration, cognition and memory of patients. Maintaining sufficient cerebral perfusion and cerebral oxygen balance is the basic safety requirement of controlled hypotension. The purpose of this study was to investigate the effects of different levels of controlled hypotension on regional cerebral oxygen saturation and POCD in patients undergoing TKA and found out the suitable range of controlled hypotension for clinical reference.

## Materials and methods

### Setting and patients

This study protocol was approved by the Medical Institutional Ethics Committee of Wei Fang People Hospital and was registered in the China Clinical Trial Center (ChiCTR2200055120). The patients, aged 55–70 years old who were diagnosed with osteoarthritis and scheduled for TKA in general anesthesia at the Wei Fang People’s Hospital From January 2022 to May 2022 were enrolled in this study. All patients had American Society of Anesthesiologists (ASA) grades I and II, and able to complete study questionnaires. All participants had to sign the informed consent form. Exclusion criteria: (1) preoperative mini-mental state examination (MMSE) score < 24 and unable to communicate normally, (2) hemoglobin (Hb) levels < 100 g/L, (3) cerebrovascular disease, (4) uncontrolled hypertension. (5) Have a neurological or psychiatric disorder.

### Randomization and blinding

Patients meeting the criteria were enrolled through preoperative visits and informed consent was signed. The patients were randomly divided into three groups by anesthetist in the preoperative, using a list of numbers generated by the QuickCalcs. The group assignment numbers were sealed in an envelope, and opened once written informed consent was obtained. In group A: MAP was maintained at 90–100% of the baseline; in group B: MAP was maintained at 80–90% of the baseline; in group C: MAP was maintained at 70–80% of the baseline. All operations were performed by the same group of surgeons, and postoperative visits were conducted by the investigator, who was unaware of the grouping.

### Anesthesia and postoperative treatment

All included patients were prepared for routing general anesthesia by inserting an intravenous line, and electrocardiogram, non-invasive blood pressure, pulse oximetry, and rSO_2_ were monitored. All patients were administered 0.5μg/kg (within 10 min) of dexmedetomidine as a premedication, and 30 mL of 0.3% ropivacaine was used for ultrasound-guided femoral nerve block. Induction anesthesia was achieved with propofol (closed loop target controlled infusion under bispectral index (BIS) monitoring, initial target concentration: 3–4 μg/mL), remifentanil (3–4 μg/kg), sufentanil (0.2μg/kg), and rocuronium (40 mg). The laryngeal mask was inserted when the patient was unconscious and BIS was stable at 40–60. The lungs were ventilated with 60% oxygen and controlled with a tidal volume of 6–8 mL/kg, and the respiration rate was 12–14 beats per minute. Propofol (closed loop target controlled infusion under BIS monitoring (40–60))and remifentanil (2.0–3.5 ng/mL TCI) was used to maintain anesthesia. Respiratory parameters were adjusted to maintain a PetCO_2_ value of 35–45 mmHg. Before surgery, target level of controlled hypotension was achieved by increasing the depth of anesthesia (remifentanil) or nitroglycerin. The norepinephrine (40μg or 0.1–0.3 μg⋅kg^–1^⋅min^–1^) would be administered to raise the MAP after lowering it below the target value. The heart rate was maintained at 50–80 beats/min and atropine and esmolol were used to regulate heart rate. All patients in the three groups maintained the MAP ≥ 70% of the baseline, and MAP ≥ 55 mmHg. When rSO_2_ < 80% of the baseline, or the lowest rSO_2_ value < 50% of the baseline and lasted more than 10 s, the blood pressure was increased until rSO_2_ returned to 80% of the baseline.

### Data collection

Before operation, relevant data was collected including gender, BMI, Hb, CRP, MMSE score, etc. The MAP, HR and rSO_2_ of baseline (T_0_), admission (T_1_), oxygen inhalation (T_2_), anesthesia induction (T_3_), 5 min after controlled hypotension (T_4_), 10 min after controlled hypotension (T_5_), 20 min after controlled hypotension (T_6_), the end of operation (T_7_) and 5 min after operation (T_8_) were observed and recorded. Postoperative Hb, CRP after operation, MMSE score at 1, 3, and 7 days after operation and PACU residence time were recorded.

### Statistical analysis

In this study, SPSS 25.0 software (developed by IBM Corp.) was used for all data processing. For continuous data, normally distributed data is expressed as mean ± standard deviation, while non-normally distributed data is expressed as median [range between quartiles (IQR)]. ANOVA was used for continuous data consistent with normal distribution and homogeneity of variance; otherwise, Kruskal-Wallis test was used. The categorical data were represented in frequency and proportion, and analyzed using the χ^2^-test or Fisher Freeman-Halton test as appropriate. *P* < 0.05 was considered statistically significant.

## Results

Forty- eight patients were initially enrolled in this study. One patient in group A was later excluded due to loss of data; one patient in group B was excluded due to the shift of brain oxygen sensor in his forehead resulting in abnormal rSO_2_ reading during controlled hypotension, which was not effective after many adjustments and both patients recovered well after operation. Finally, forty-six patients were enrolled.

### Baseline characteristic

No significant differences in the baseline characteristic data were observed between the three groups, such as sex, age, BMI, ASA classification (Table 1).

**TABLE 1:** Demographic and preoperative characteristics.

	Group A	Group B	Group C	*P-*value
Male, *n* (%)	11(73.3%)	11(68.7%)	11 (73.3%)	0.947
Female, *n* (%)	4(26.7%)	5(31.3%)	4(26.7%)	
Age (year)	60.87 ± 3.93	61.06 ± 3.71	62.60 ± 4.14	0.606
BMI (kg/m^2^)	26.18 ± 2.40	27.20 ± 3.74	26.07 ± 2.59	0.514
ASAI, *n* (%)	2(13.3%)	2(12.5%)	3(20.0%)	0.819
II, *n* (%)	13(86.7%)	14(87.5%)	12(80.0%)	
Preoperative MMSE score	28.07 ± 1.34	28.19 ± 1.17	27.33 ± 1.11	0.119
Hemoglobin (g/L)	132.53 ± 12.16	128.70 ± 9.68	126.35 ± 8.91	0.264
CRP (mg/L)	3.50 (2.40, 5.60)	2.90 (1.53, 4.78)	2.40(1.50, 6.20)	0.672

Values are means ± SDs, numbers (%), or medians and ranges. BMI, body mass index; ASA, American Society of Anesthesiologists; CRP, C-reactive protein.

### Primary outcome

#### Influence of controlled hypotension on rSO_2_

**FIGURE 1:**
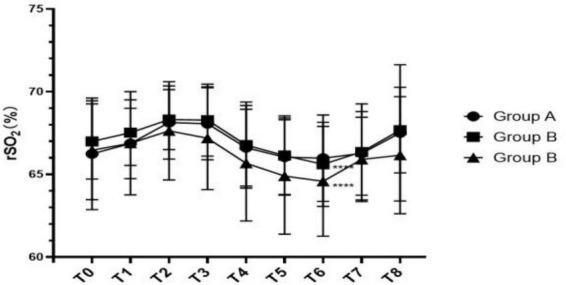
Line chart of rSO_2_ changes at different time points.

As is shown in Table 2, there was no statistical difference in rSO_2_ between groups at each time point, but there was significant statistical difference at each time point within the Group B and Group C (Group B: *P* = 0.021; Group C: *P* = 0.028). The change trend of rSO_2_ at each time point is shown in Figure 1. Before T3, the tendency of rSO_2_ in all group was upward, then it began to descend after controlled hypotension. The rSO_2_ in Group C was lower than that in the other two groups between T2-T8, but there was no statistical difference. There are relatively clear downward trend of rSO_2_ in the Group B and Group C, and the influence of blood pressure on rSO_2_ was more obvious at T6.

**TABLE 2:** Changes in rSO_2_ at various time points among the three groups.

	T_0_	T_1_	T_2_	T_3_	T_4_	T_5_	T_6_	T_7_	T_8_	*P*	*F*
Group A	66.24 ± 3.37	66.87 ± 2.13	68.13 ± 2.21	68.06 ± 2.18	66.61 ± 2.32	66.04 ± 2.26	65.98 ± 2.61	66.27 ± 2.53	67.51 ± 4.12	0.156	1.521
Group B	66.99 ± 2.27	67.52 ± 1.98	68.31 ± 1.81	68.28 ± 2.18	66.77 ± 2.60	66.14 ± 2.40	65.61 ± 2.54	66.36 ± 2.91	67.67 ± 2.59	0.021	2.358
Group C	66.46 ± 2.99	66.88 ± 3.12	67.63 ± 2.97	67.18 ± 3.11	65.67 ± 3.49	64.90 ± 3.51	64.58 ± 3.32	65.90 ± 2.55	66.16 ± 3.54	0.028	2.246
*p*-value	0.764	0.710	0.724	0.453	0.517	0.372	0.379	0.882	0.431		
*F*	0.85	1.085	1.025	2.530	2.106	3.176	3.115	0.394	2.694		

**FIGURE 2:**
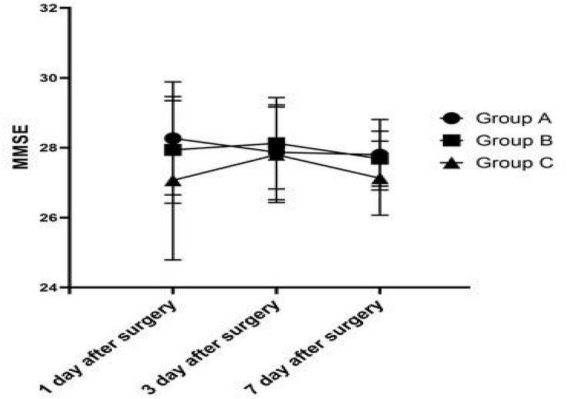
Comparison of postoperative MMSE scores among the three groups.

We further analyzed the correlation between the percentage of hypotension amplitude in each group and the percentage of rSO_2_ reduction at three time points in the controlled hypotension process (Figure 2 and Table 3), but none of the differences were statistically significant (*p* = 0.417). It is not difficult to find that the possibility of rSO_2_ decline in group C is relatively large.

**TABLE 3:** The decreases in MAP and rSO_2_ during controlled hypotension in all groups.

	Group A	Group B	Group C	*P*
△MAP	6.86 ± 3.78	16.60 ± 3.12	24.42 ± 5.37	<0.001
△rSO_2_	–0.29 (–3.28, 3.19)	1.03(–3.21, 3.69)	0.61 (–0.70, 5.36)	0.417

#### Influence of controlled hypotension on cognitive function

The cognitive function on 1, 3, and 7 days after surgery was measured using MMSE. There was no statistically significant difference (1 day after surgery: *P* = 0.192; 3 day after surgery: *P* = 0.777; 7 day after surgery: *P* = 0.136) between groups (Figure 2). The MMSE score of Group C was always lower than that of Group A and B after operation.

### Secondary results

#### Intraoperative medication

Among all intraoperative drugs, only remifentanil and deoxyepinephrine showed significant statistical difference(remifentanil: *P* < 0.001, deoxyepinephrine: *P* < 0.001) (Table 4). However, the use of propofol, atropine and nitroglycerin among the three groups has no significant difference.

**TABLE 4:** Comparison of intraoperative medication among the three groups.

	Group A	Group B	Group C	*P*-value
Remifentanil (mL, 20 μg/mL)	11.65 ± 0.93^a^	12.63 ± 1.50^b^	14.48 ± 1.72^ba^	<0.001
Propofol (mL, 10 mg/mL)	37.40 ± 2.16	36.10 ± 5.60	37.93 ± 4.83	0.532
Deoxyepinephrine (μg)	100.00 (60.00, 140.00)^dc^	20.00 (0.00, 70.00)^c^	0.00 (0.00, 0.00)^d^	<0.001
Nitroglycerin (μg)	0.00 (0.00, 0.00)	0.00 (0.00, 0.00)	0.00 (0.00, 20.00)	0.057
Atropine (mg)	0.00 (0.00, 0.00)	0.00 (0.00, 0.00)	0.00 (0.00, 0.00)	1.000

^a^Group A vs. group C, *P* < 0.001, ^b^Group B vs. group C, *P* = 0.003, ^c^Group A vs. group B, *P* = 0.022, ^d^Group A vs. group C, *P* < 0.001.

**FIGURE 3:**
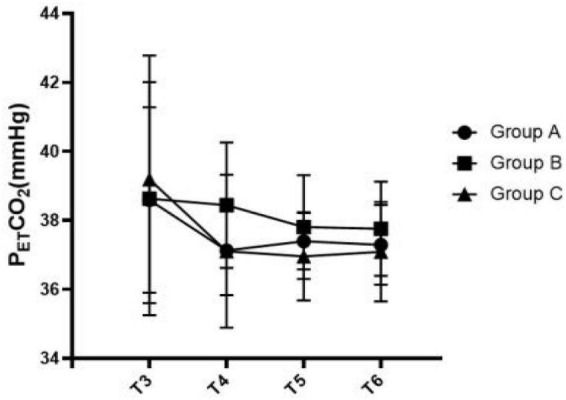
Comparison of P_KT_CO_2_ at various time points among the three groups.

**FIGURE 4:**
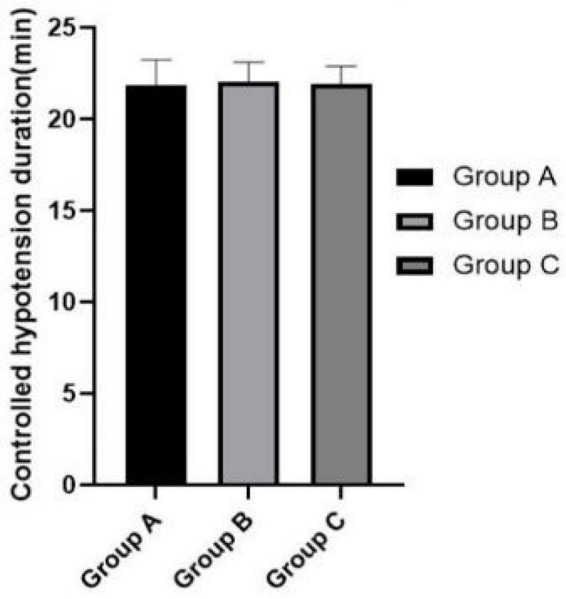
Comparison of controlled hypotension duration.

**FIGURE 5:**
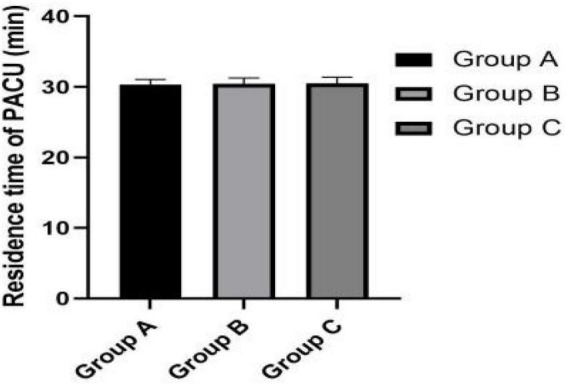
Residence time of PACU.

**FIGURE 6:**
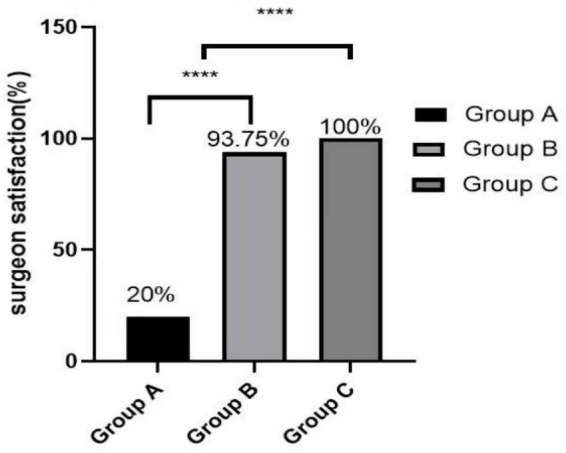
Surgeon satisfaction. ****Group A vs. Group B, *P* < 0.001 and Group A vs. Group C, *P* < 0.001.

Figures 3–5 show that the P_ET_CO_2_, controlled hypotension duration and residence time of PACU were similar between these groups (*P* > 0.05). Figure 6 shows that Surgeons’ satisfaction increased significantly in groups B and C (*P* < 0.001), and the magnitude of blood pressure reduction in Group A was hardly satisfactory to the doctor.

#### Bleeding and inflammation

Among the three groups, although there was no significant difference in the percentage decrease of hemoglobin level (*P* = 0.935), there was significant difference in postoperative CRP levels (*P* < 0.001) (Table 5). The results shown in Table 5 indicate the percentage reduction of hemoglobin in Group C was the least compared to the other two groups. The CRP index in Group A was significantly higher than that in the other two groups (Group A vs. group B, *P* = 0.003; Group A vs. group C, *P* < 0.001).

**TABLE 5:** Comparison of postoperative indicators among the three groups.

	Group A	Group B	Group C	*P*-value
Percentage decrease of hemoglobin level	0.15 ± 0.04	0.15 ± 0.04	0.14 ± 0.03	0.935
CRP (mg/L)	108.00 (89.00, 168.00)^ab^	73.45 (18.20, 82.60)^a^	32.50 (19.10, 58.10)^b^	<0.001

^a^Group A vs. group B, *P* = 0.003; ^b^Group A vs. group C, *P* < 0.001.

## Discussion

A total of 46 participants were included in this study. We found that the short-term (rSO_2_) or relatively long-term effects (MMSE score) of the magnitude of blood-pressure reduction in the three groups were limited, and there was some trend but no significant statistical difference. This difference may be made more pronounced by enlarging the sample size. Another finding is that the differences in primary outcomes among the three groups increased with the duration of hypotension.

Severe intraoperative and postoperative blood loss caused by TKA has always been a concern (3). Controlled hypotension technique and tourniquet are usually used to reduce intraoperative bleeding during TKA. Studies have shown that the use of tourniquet during operation will cause tourniquet-related ischemia-reperfusion injury (4). Some studies also found that the patients who experienced controlled hypotension but without tourniquet use during the operation had higher MOCA scores than those patients who used tourniquets (5). Therefore, we used controlled hypotension to reduce bleeding in this study.

Controlled hypotension is defined as the reduction of systolic blood pressure (BP) to 80–90 mmHg, and that of MAP to 50–65 mmHg or 30% lower than the baseline level (1) while still providing adequate oxygen delivery to vital organs. A research has shown that lowering the MAP to 2/3 of the initial value does not cause any damage (6, 7). Therefore, we set the 30% decrease from the baseline MAP as the minimum levels to investigate the effects of different levels of controlled hypotension on regional cerebral oxygen saturation and postoperative cognitive function in patients undergoing TKA.

However, one study had shown that extreme hypotension may cause organ insufficiency and subsequent ischemic injury of vital organs, especially the brain (2), which has potential risk to cognitive function.

The biggest risk of controlled hypotension is hypoxic brain damage caused by insufficient cerebral blood perfusion. rSO_2_ mainly monitors the oxygenation at the level of bilateral frontal lobes, but it can roughly reflect the balance of oxygen supply and demand in the whole brain. In this study, we aimed to determine the relationship between the different degrees of controlled hypotension and postoperative cognitive function, Studies have shown that intraoperative hypotension is a risk factor for POCD (8–10). However, the conclusion was not reached in our study, we observed that a 20–30% decrease of baseline MAP allowed reduction of bleeding in the surgical field, minimization of blood loss, better visibility and therefore, it increases the surgeon’s comfort, reduces the surgery time and prevents complications. An analytical research about risk factors of early cognitive dysfunction in Elderly Patients after Total Knee shown that the level of preoperative cognitive function, postoperative CRP level, and postoperative pain were independent risk factors for POCD (11). However, no significant differences in the baseline characteristic data were observed between the three groups, such as sex, age, BMI, SBP, DBP, Hb levels, CRP levels, and ASA physical status, even the baseline MMSE score.

In this study, we adjusted the depth of anesthesia by adjusting the dose of remifentanil. When the expected hypotension level was not reached, we combined nitroglycerin to reduce the blood pressure to the target value. Remifentanil (RFN), an ultrashort-acting opioid and μ receptor agonist, enables easy adjustment of the depth of anesthesia and reduction of the MAP and HR through cardiodepressive action (12, 13). Earlier studies also indicated the positive effect of propofol as an anesthetic in reducing MAP through its effect on precapillary arterioles (14). In this study, we used propofol under closed-loop systems under BIS anesthesia depth monitoring to eliminate the effect of propofol on POCD. In addition, the research of Picton P shown that CO_2_ levels can influence CBF by regulating the dilation and constriction of cerebral vessels and alter rSO_2_ levels (15), and this result is also consistent with some other studies, they also found the correlation analysis between PetCO_2_ and rSO_2_ (16, 17). Therefore, we kept the P_ET_CO_2_ in the three groups at a similar level.

Our results showed that MAP values decreased in all groups immediately after induction of anesthesia (T_3_), but instead of decreasing with it, the value of rSO_2_ rised, and this may be related to the inhalation of 100% oxygen. During controlled hypotension, we found that rSO_2_ gradually decreased and reached the lowest at T_6_ with the continuation of controlled hypotension, while the value was restored to the baseline level at T_7_ and T_8_. However, although the three groups had different degrees of controlled hypotension, there was no statistical difference in rSO_2_ at the same time among the three groups. Some studies found that MAP was moderately cross-correlated with current rSO_2_ (*r* = 0.728) (9, 18), but our study is not consistent with it. From the analysis of our results, it can be seen that the decrease of rSO_2_ has the same trend as the decrease of MAP during the controlled hypotension period, however, we didn’t find the significant correlation between the decrease percentage of rSO_2_ and MAP in the three groups. When the baseline MAP decreased by 20–30% (24.42 ± 5.37), the decrease in rSO_2_ from the baseline was only 0.61 (–0.70, 5.36)% in Group C. At the same time, analysis of cognitive function on 1, 3, and 7 days after surgery showed that MMSE scores were lowest in the group with the maximum degree of controlled hypotension, although there was no statistically significant difference and none of the patients had obvious clinical manifestations of cognitive dysfunction. This may be related to the short duration of controlled hypotension and surgery in this study. Some studies also revealed that longer duration of surgery (OR1.82, 1.01∼3.16) were risk factors for POCD (19). A randomized trial shown that BIS decreases delayed neurocognitive recovery and postoperative neurocognitive disorder (20). And preoperative use of dexmedetomidine (OR0.70, 0.08∼0.94) and preemptive analgesia (OR0.75, 0.13–0.90) were the protective factors for POCD in elderly patients with laparoscopic surgery (12). In our study, the maximum degree of controlled hypotension is 30% of the baseline MAP, and the minimum value of MAP is above 60 mmHg. In brain physiology, cerebral pressure autoregulation, during MAP between 60 and 150 mmHg, maintains a relatively stable CBF. Since the reduction from baseline MAP did not proceed beyond the range of cerebral autoregulation, the grouping of this study is relatively reasonable, and both MAP and rSO_2_ values were restored to baseline levels after the end of the operation. The results of a study showed that mean arterial pressure < 50 mmHg (1 mmHg = 0.133 kPa) was closely related to a decrease in cognitive function (21).

A Randomized Controlled Trial about the effect of deliberate hypotension on regional cerebral oxygen saturation during functional endoscopic sinus surgery shown that none of the patients experienced cerebral desaturation events (CDEs) when the baseline MAP was decreased by 30%, which result is also consistent with our study (9). In this study, monitoring of rSO_2_ while controlled hypotension and timely intervention can reduce the occurrence of postoperative neurological disorders, and the criteria for cerebral ischemia are reduction of 10 index points in rSO_2_ from a stable baseline, absolute value of rSO2 < 50%, 20–25% reduction in relative rSO_2_ (22). Thus, rSO_2_ was more than 20% lower than the baseline, the controlled hypotension was stopped in the event of further drop till rSO_2_ was restored to acceptable levels.

The conclusion that the occurrence of cognitive dysfunction is affected by intraoperative blood loss including postoperative oozing in elderly patients has consistently been confirmed (8, 9). Studies have shown that the estimation of hemoglobin mass loss was found to be a more accurate method to estimate perioperative blood loss (23). In our study, percentage decrease of hemoglobin level is least in Group C, although no statistically significant differences were observed among the three groups. Moreover, Group C obtained the best surgical field and surgeon satisfaction. In addition, there was no statistical difference in PACU residence time among the three group. More severe controlled hypotension does not present a disadvantage in postoperative recovery.

A finding indicated that inflammation is significantly associated with the development of POCD. In molecular biology, microglia are important cells that maintain the balance of inflammation in the brain (24). The increased levels of peripheral inflammatory factors induced by surgical stimuli cause microglial activation (25), which produce cytokines and result in the production of a range of inflammatory factors in areas of the central nervous system (CNS). However, there are researches finding that the mechanism of POCD is related to inflammatory response (26, 27), CRP serves as a marker of non-specific acute-phase response in inflammation, infection, and tissue damage, related to the development of POCD. Several studies demonstrated that increased CRP levels were associated with POCD (23, 28–30). In our study, CRP in the group C was significantly lower than that in the other two groups, but none of the patients developed POCD.

This study has some limitations. First, the groups in this study were too small to achieve statistical significance when they were divided into subgroups, and we suggest that further studies should incorporate larger sample sizes. Second, we used MMSE as a measure for cognitive function, which is a very quick cognitive test, but is less suitable to detect the subtle cognitive impairment associated with POCD (31). Third, the patients we selected had an limit of age, partly because the study population was relatively young, and the average age was 62.51 ± 3.89 years. Therefore, the extrapolation of these results to elderly surgical patients must be cautious, because these elderly patients have become more sensitive to the development of POCD itself, even without hypotension. Finally, in this study, the lowest value of the controlled hypotension is only 30% of the basic value, we can try to further reduce the degree of the controlled hypotension to observe its relationship with rSO_2_.

## Conclusion

Our study found that the decrease of rSO_2_ was related to the decrease level of MAP in TKA. Based on our findings, we recommend that the controlled hypotensive target indicated by MAP be reduced by 20–30%, which not only decreases intraoperative bleeding and improves the quality of the surgical field of vision, but also is still within safe levels.

## Data availability statement

The original contributions presented in this study are included in the article/supplementary material, further inquiries can be directed to the corresponding author.

## Ethics statement

The studies involving human participants were reviewed and approved by the Medical Institutional Ethics Committee of Wei Fang People Hospital. The patients/participants provided their written informed consent to participate in this study.

## Author contributions

YZ: manuscript writing. CZ: collecting data. SR: statistical analysis. JF, NL, and ZZ: literature review. BL: design research plan. All authors contributed to the article and approved the submitted version.
